# Development of a 3D transparent aortic model as a radiation-free training simulator for basic skills of endovascular aortic interventions

**DOI:** 10.1186/s12909-025-08422-x

**Published:** 2025-12-09

**Authors:** Ahmed Ghazy, Husain Jaber, Mohamed Albitar, Philipp Pfeiffer, Jan Beer, Marta Medina, Hendrik Treede, Ryan Chaban

**Affiliations:** Department of Cardio-Vascular Surgery, University Hospital of Johannes Gutenberg University, Mainz, Germany

**Keywords:** Endovascular aortic repair, Medical education, 3D model, Surgical simulators, Training

## Abstract

**Background:**

Endovascular aortic repair (EVAR) are gradually replacing open surgical repair for aortic diseases, due to their minimally invasive nature. These procedures require a high level of expertise that is gained through extensive clinical experience, posing risks such as prolonged radiation exposure. Training on 3D-printed simulation models can offer safer environment for learning & improve procedural precision & outcome.

**Method:**

A single-center study to evaluate whether training on radiation-free three-dimensional (3D) printed aortic training models can reduce the time required from vascular surgeons to complete the basic endovascular navigation tasks, such as navigating the guide wire & probing the different branches. We involved 15 vascular doctors, 8 in-experienced (group1) & 7-experienced (group2). Participants received a 15-min lecture on aortic interventions and guide wire handling. Two self-made 3D printed training models were used. Models either are of the entire aorta (ascending to iliacs, see model1) or thoracoabdominal aorta (model2) were used. A covering box and a camera positioned above the aortic model that mirror the intervention on a monitor, simulating the indirect vision of the intraoperative fluoroscopy. Participants were required to probe and intubate four aortic branches (two renal arteries, coeliac trunk, & superior mesenteric artery) in three steps: initial assessment, 15-min training, & post-training assessment (next day). Task completion times were recorded & analyzed.

**Results:**

Group1 initially required 914 ± 420 s to intubate four ostia, which significantly decreased to 149 ± 48 s post-training (*p* = 0.001). Experts showed no significant time reductions following the training (*p* = 0.443). Initial times were significantly lower for Group2 (*p* = 0.002), but post-training times showed no significant difference between both groups (*p* = 0.134).

**Conclusion:**

Using 3D-printed models in a simulation-training may help to familiarize & train participants with endovascular aortic procedures within limited material costs, leading to significant reductions in task completion time among trainees. Additionally, a significant reduction in simulated visualization time was observed, suggesting potential for reduced fluoroscopy exposure in real procedures.

**Trial registration:**

The institutional ethics committee of the University Hospital of Mainz approved this study.

## Background

Endovascular aortic repair (EVAR) have emerged as the optimal therapeutic option for a variety of aortic diseases, progressively supplanting many traditional surgical approaches [1]. these interventions, however, demand a high level of expertise and precision, which can only be achieved through extensive clinical experience. The conventional methods of acquiring such experience in clinical settings pose significant challenges, primarily due to patient safety concerns, as excessive manipulation of the diseased aorta can results in complications (bleeding, dissection, etc.) and the risks associated with prolonged radiation exposure on both the patient and the medical staff [2].

To mitigate these risks, appropriate transparent simulation models have been developed, offering a valuable opportunity for medical professionals to train and gain the necessary experience with minimal risk [3]. These models enable practitioners to improve their skills in a controlled environment, ensuring patient safety and reducing the potential hazards associated with real-life procedures [4].

The integration of three-dimensional (3D) technology into the medical field is witnessing rapid growth [5]. This technology is utilized in various capacities, including the planning of complex interventions [6], the creation of 3D-printed prostheses [7], and the development of anatomically accurate simulators [8]. The use of 3D printed simulation models enhances the precision of aortic interventions and facilitates the training process, allowing for a more thorough and practical learning experience [9].

The aim of this study is to evaluate whether training on home-made radiation-free 3 d printed aortic training models can reduce the time required from vascular doctors to complete the basic endovascular navigation tasks, such as navigating the guide wire and probing the different branches.

## Methods

### Study design

Between February 2023 and June 2024, in a single-center study we involved15 vascular doctors were enlisted: 8 beginners (Group 1, < 5 years of independent experience, < 25 Intervention) and 7 experts served as the control reference representing the level to be achieved by the inexperienced participants in case of any potential skill development (Group 2, > 5 years of independent experience, > 100 Intervention). Time was used as a measurable parameter. All participants received an introductory lecture for 15 min on aortic interventions and the handling of the aortic guide wires and catheters.

Two 3D training models were used. Each model consisted of I) Models either are of the entire aorta (ascending to iliacs, see model 1) or thoracoabdominal aorta (model 2) were used, both models were developed and printed using the 3D printers available in the university of Mainz, The models were printed using VeroClear™ (Stratasys Ltd., Minnesota, USA), a transparent rigid photopolymer. After printing, the support material (SUP706) was removed by water jet and sodium hydroxide bath, a manual removal of supporting structures and polishing was done to achieve the needed transparency. II) a covering box for the models was used with a visible III) port (7 Fr.) access to the “common iliac artery” and IV) a camera installed vertically in a position simulating the X-ray tube and mirroring the intervention on V) a liquid–crystal display monitor (Fig. 3), to simulate the indirect vision of the intraoperative fluoroscopy.

**Fig. 1:**
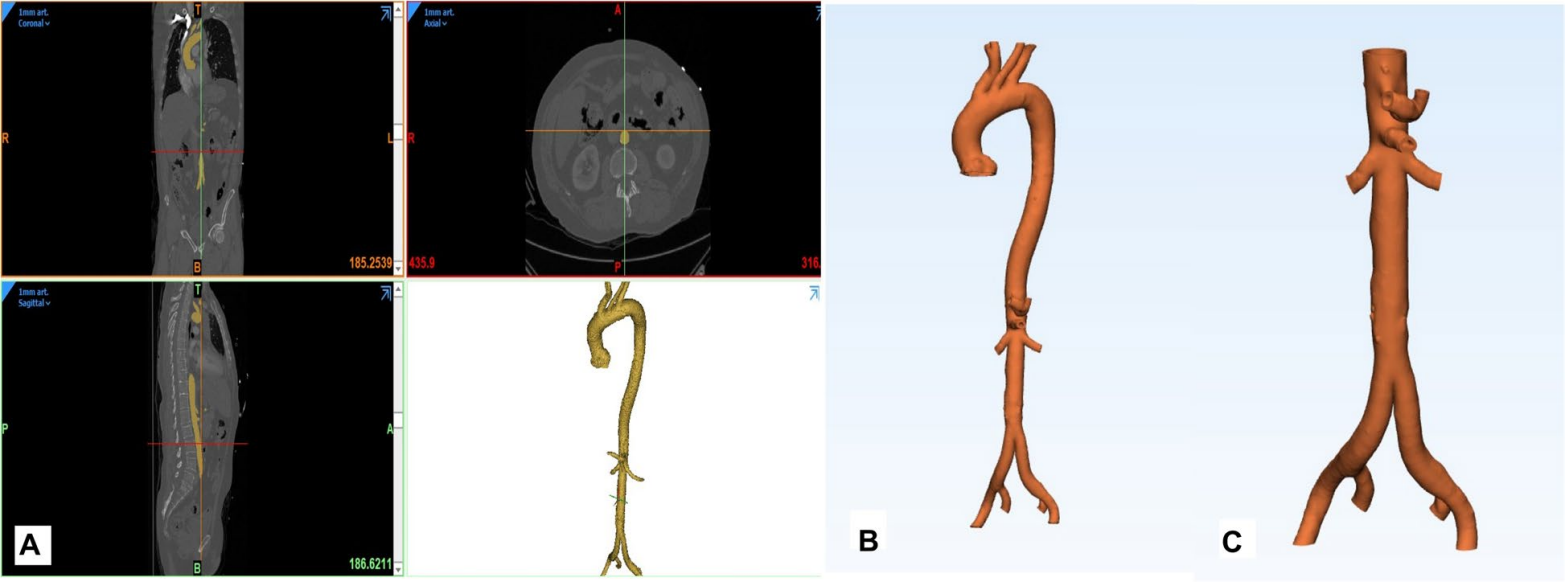
Generation of the STL-file. **A** With the Mimics Innovation Suite (MIS) software package, the blood volume (aorta and iliac bifurcation) was segmented from the original DICOM dataset (resolution 1 mm) and transformed into an STL-file. The STL-file of the blood volume was then imported into the 3Matic-module of MIS. **B** Model 1 of the whole aorta was generated Using the “hollow”-tool, a wall thickness of 2 mm was applied externally to the aortic blood volume. **C** Model 2 for the part of interest (abdominal aorta) was generated. The ostia were opened using the “trim”-tool and both models were exported as STL-file

**Fig. 2:**
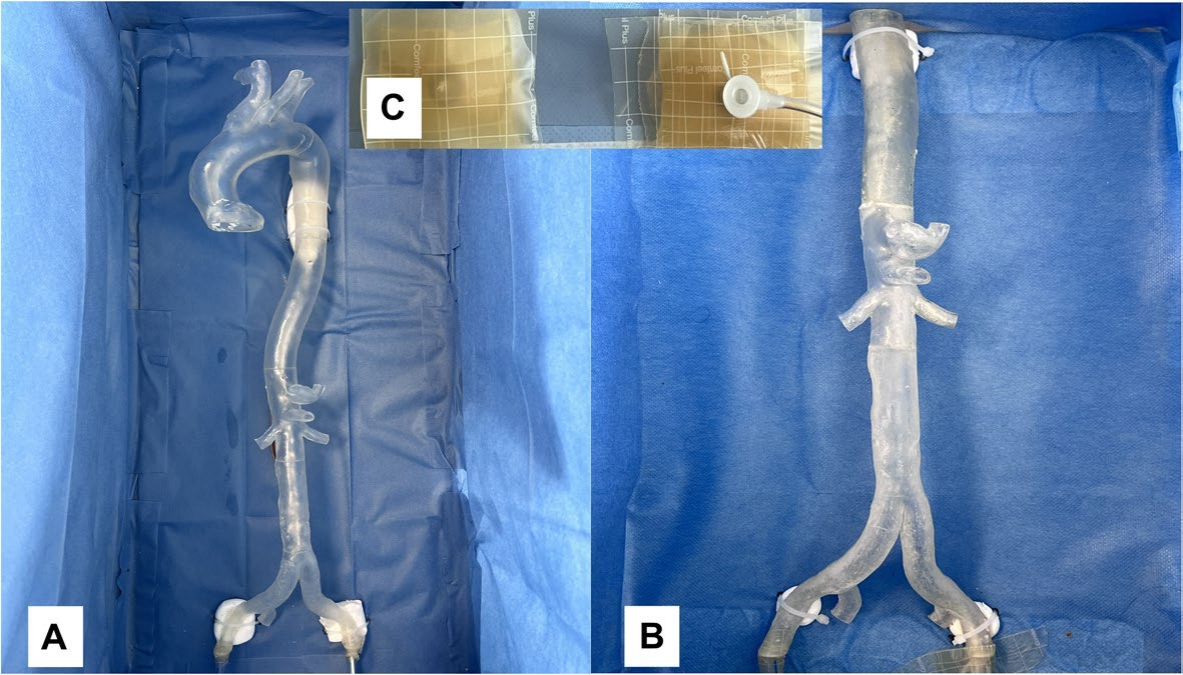
**A** 4 Parts of Model 1 glued together **B** 3 Parts of Model 2 glued. Both Models were fixed in the covered box, leaving only the femoral access accessible through art. 5 Fr. Sheath. **C** Showing the femoral access from outside

Through the following steps, A 3D post-processing software (Mimics Innovation Suite, Materialise, Belgium) was used to segment the Aorta from anonymized datasets of representative computed tomography (CT) scans performed in our institution. First, the aortic blood volume was segmented on CT images with the low to high intensity threshold set from 280 to 740 Hounsfield units (HU) respectively (Fig. 1). Afterwards, using the ‘Hollow’ tool of the design software (3-Matic, Materialise, Belgium, Fig. 1). An aortic wall with a thickness of 2 mm was added to the aortic blood volume. The aortic branches were trimmed to a length of 2 cm. Finally, a 3D file (.stl) was generated and printed on an PolyJet 3D printer (Objet Prime 30, Startasys, Minnesota, USA) using the Polyjet photopolymer. The 3D model was printed in 4 parts for Model 1 and 3 Parts for Model 2. The added supporting structures were removed at the end. Using Objetstudios-software. The positioning of the model in the printer, generation of support structures and determination of the level of printing quality (High-quality) were performed. At the end, the model was processed to reach a high degree of transparency and the different model parts were glued to each other’s (Fig. 2).

### Experimental protocol

**Fig. 3:**
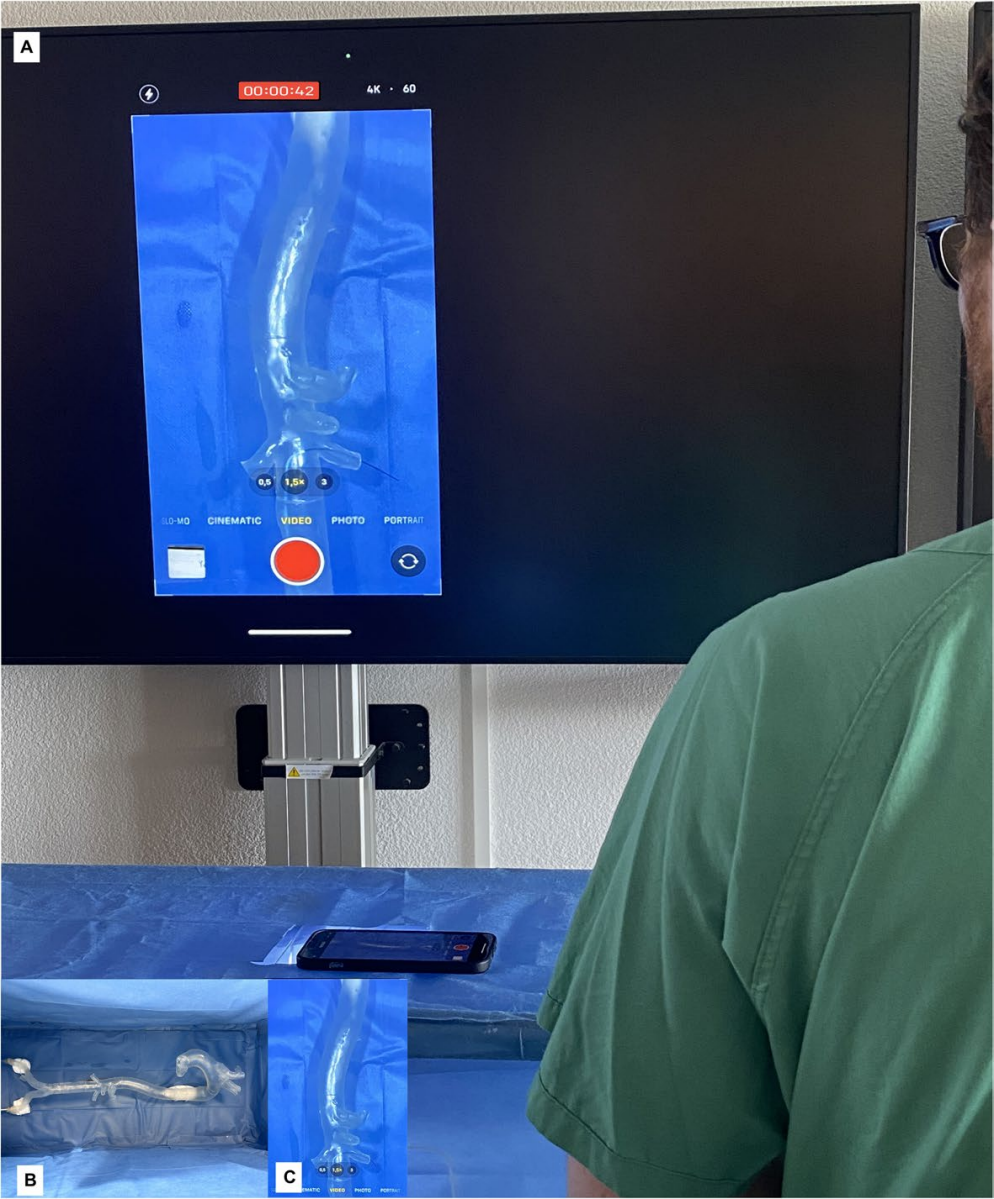
**A** The Experimental setting showing a transparent 3-D printed aortic model of the aorta placed in a box and covered, with a camera fixed on the box and the image mirrored on the screen simulating the fluoroscopy settings in the operative theatre. Using a Bluetooth connected device, every participant could activate/deactivate the camera, and the time needed was recorded. **B** Insert: view inside box with aortic model inside the box. **C** A closer view of the camera image with the guiding wire introduced in the left renal artery using a multi-purpose catheter

Participants were required to probe and intubated four aortic branches (two renal arteries, coeliac trunk, and superior mesenteric artery) in three steps:

For the experiment, the 3D printed model was placed and fixed on a box and covered with a surgical drape leaving only the common iliac artery accessible. During the intervention, a camera was placed over the box and a screen mirroring was performed on a liquid–crystal display monitor (Fig. 3) to simulate the intraoperative fluoroscopy procedure. The time needed to complete the different tasks was recorded (effective needed time). Simultaneously, every participant had to activate/deactivate the camera by pressing on a bluetooth connected Button/Pedal to the camera and the time used by participant was recorded (visualization time/simulated radiation time).

**Fig. 4:**
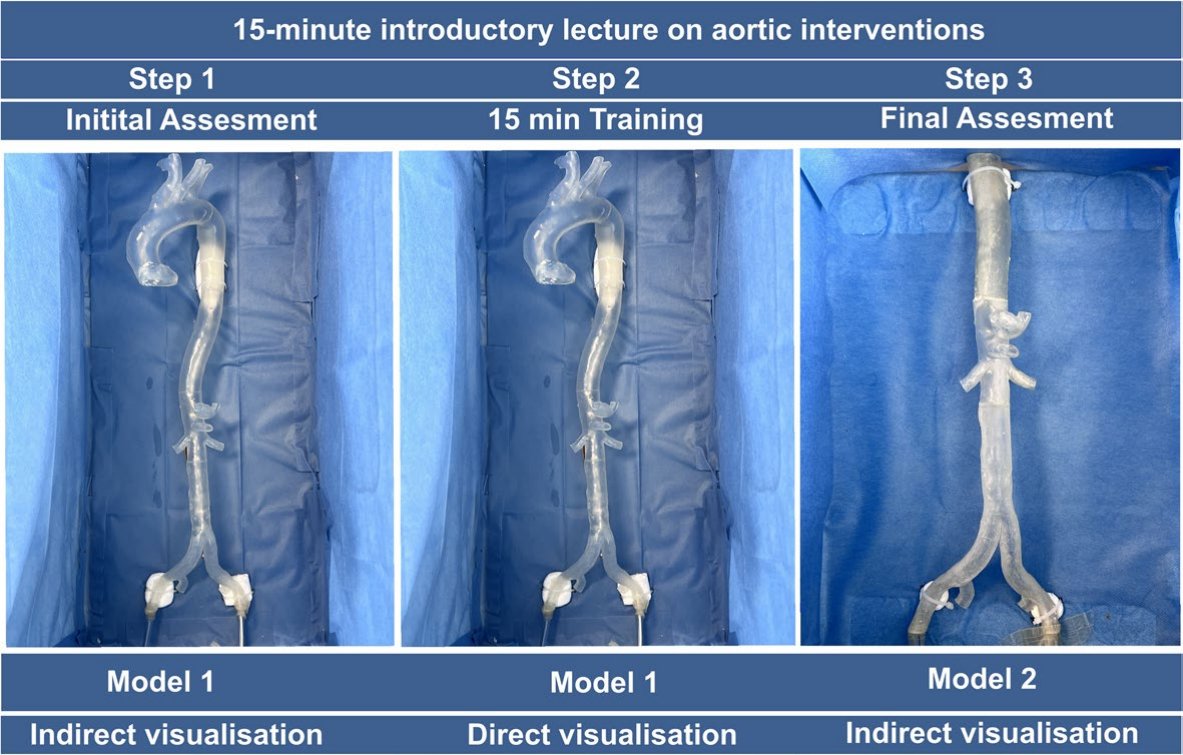
Experimental protocol with introduction (15 min) followed by experimental Step 1 (initial assessment), Step 2 (15-min training period) and Step 3 (post-training assessment). For Steps 1 & 2 the model 1 was used, for Step 1 with indirect visualization and Step 2 with direct visualization. For Step 3 a different model (Models 2) was used with indirect visualization

We adopted a protocol for each participant: 4 given ostia (two renal arteries, coeliac trunk, and superior mesenteric artery) were intubated in a three-step design of initial assessment on Model 1 with indirect visualization (Step 1), a training period of 15 min on Model 1 (Step 2) with direct visualization of the model. A post-training assessment on Model 2 with indirect visualization of the procedure on a monitor (Step 3). Of note, to minimize adaption, the assessment step was performed on the next day and on a different 3D model. For the 1 st step, each participant had to intubate a set of four ostia as mentioned above with random order that was assigned by the principal investigator. During the training period, each participant could perform repetitive intubations as needed. For the 3rd step, again, each participant chose by lot a new order of the above mentioned 4 tasks to intubate on model 2 and the time was recorded again. After each attempt, the guidewire was retracted into the aorta, and the investigator started time recording once the next intubation trial began (overall effective time). Separately, the visualization Time was recorded by every participant (Fig. 4).

### Statistical analyses

Statistical analysis was done with Prism 10 (GraphPad software, MA, USA). For continuous variables, a mean and a standard deviation were calculated. Normality analysis for continuous variables using the Shaprio-Wilk Test was performed. In case of normal distribution, the paired t-test was used. Otherwise, a Mann–Whitney-U test was performed.

*P* < 0.05 was considered significant (*) and *P* < 0.001 was considered highly significant (**). No adjustment for multiple testing was applied.

## Results

**Fig. 5:**
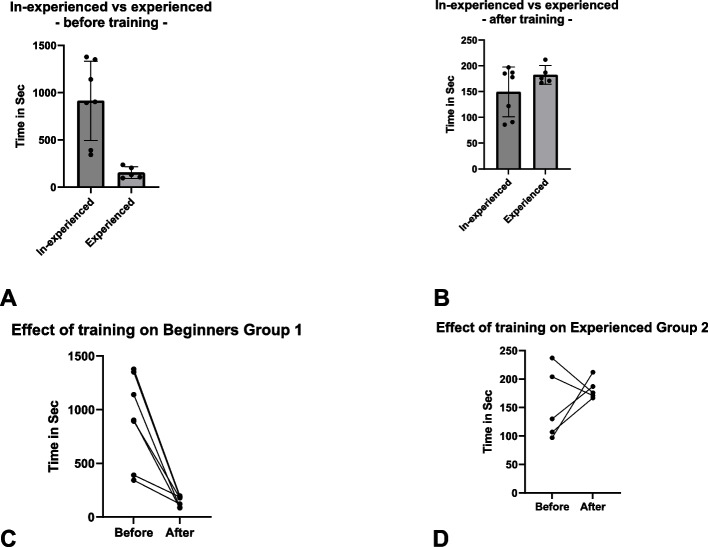
**A** and **B** Overall results for Steps 1 (**A**) and Step 3 (**B**) of both groups are depicted (data are shown as mean ± standard deviation). **C** and **D** Results of the overall time of Steps 1 (Before) and Step 3 (After) for both groups are demonstrated in seconds

The overall effective time needed for inexperienced (group 1) to intubate 4 randomly assigned ostia was 914 ± 420 s (Step 1). After a training period (step 2), we noted a significant reduction in the time needed to complete the tasks in step 3 to 149 ± 48 s (*p* = 0.001). For the experienced (group 2), the overall time to complete step 1 was 155 ± 62 s, However, no significant difference was observed when compared to the post-training results (step 3) in this group with 183 ± 18 s (*p* = 0.22). The time needed to complete step 1 was significantly lower in the experienced (group 2) when compared to the in-experienced (group 1) (*p* =.0.003). However, post-training comparison (step 3) no difference between both groups was observed (*p* = 0.134) (Fig. 5).

Similar findings were observed in the comparison of the visualization time needed with a mean of 950 ± 414 s in group 1 and 171 ± 69 s in group 2 (*p* = 0.003) before training. After training no significance was observed between both groups.

When looking at the different times for each of the four separate tasks in step 1, it became evident that in group 1 there was a significant reduction from the initial 136 ± 107 and 442 ± 116 s needed to introduce the guide wire in the left renal artery and the coeliac trunk of the 3D Model to 29 ± 7 and 61 ± 25 s needed to complete the same task after the training period (*p* = 0.018 and < 0,001 respectively).

In the comparison between groups for the individual tasks, a significant difference (*P* = 0.03 and < 0.001) was observed between the two groups before the training period in the task 2 (left renal artery) and task 4 (Coeliac trunk). After the training, the reduction of time observed in group 1 succeeded in resolving the difference between the two groups (Table 1).

**Table 1:** A demonstration of the different times recorded in seconds from both groups

			**Step 1**						**Step 3**				
***Tasks***		**Renal art. (right)**	**Renal art. (left)**	**Superior mesentric art**	**Coeliac Trunk**	**Overall Time**	**Visualization Time (Simulated Radiation Time)**	**Renal art. (right)**	**Renal art. (left)**	**Superior mesentric art**	**Coeliac Trunk**	**Overall Time**	**Visualization Time (Simulated Radiation Time)**
*Group 1 (inexperienced)*	Mean	166	136	170	442	914	950	32	29	28	61	149	159
	*P*value (Before vs After Training)							0,05^*^	0,02^*^	0,04^*^	< 0,001^**^	0,001^**^	< 0,001^**^
*Standard Deviation*		188	107	175	116	420	414	13	7	7	25	48	47
*Range*		32–440	23–270	9–500	270–540	343–1378	387–1397	16–48	19–36	18–39	22–80	86–197	94–202
*Group 2 (experienced)*	Mean	34	30	23	68	155	171	43	38	38	63	183	182
	*P*value (Before vs After Training)							0,16	0,15	0,15	0,41	0,22	0,4
*Standard Deviation*		22	11	9	33	62	69	23	11	22	19	18	29
*Range*		12–65	13–43	14–33	34–106	97–237	78–254	16–78	30–57	18–75	34–80	167–212	131–219
*Pvalue*		0,043^*^	0,04^*^	0,07	0,003^*^	0,003^*^	0,003^*^	0,4	0,28	0,36	0,96	0,13	0,3

*P* < 0,05 was considered significant (^*^) and *P* < 0,001 was considered highly significant (^**^)

## Discussion

In summary, the adoption of 3D technology in medical practice, particularly in the context of aortic interventions, offers substantial benefits. These include enhanced training opportunities, improved procedural planning, and the development of customized prosthetic devices. As this technology continues to evolve, its applications are expected to expand, further transforming the landscape of medical care and intervention [10].

In our study, we observed a significant reduction in the time needed for our in-experienced group of participants after a training period. Moreover, inexperienced participants demonstrated significant improvement in procedural needed time during our training algorithm that rendered them equally to experienced participants for introducing the guidewire in the different delegated tasks.

This finding in reduction of task completion times among inexperienced group highlights the advantage of 3D-printed models to improve procedural familiarity of basic procedural skills. This is also consistent with existing literature, which emphasizes that simulation-based training can bridge the gap between theoretical knowledge and practical expertise, fostering a safer and more efficient learning environment [11, 12]. This is particularly crucial in the context of aortic interventions, where longer time means a prolonged radiation time which can be harmful for both the patient and the medical stuff [13].

Moreover, the use of 3D printing technology allowed us to create anatomically accurate models of the aorta. This precision is critical for effective training, as it ensures that participants are practicing on models that closely mimic real human anatomy [14]. Using two different models on two different occasions allowed us to reduce the adaption that could be acquired in case of the traditional simulators. The ability to customize these models based on patient-specific anatomy further enhances their utility, enabling tailored training scenarios that can address a wide range of clinical situations within an affordable limit of costs [15].

Our findings suggest that incorporating 3D-printed models into the curriculum of vascular training programs could enhance the quality of education. Using a structured training algorithm, the 3D-printed models could serve as a supplementary tool within vascular training programs [16]. However, a further validity framework is needed for this integration [17]. Furthermore, the economic advantage of 3D-printed models (< 500 euros) over traditional simulation tools (1000–5000 euros) [17] makes this approach accessible and scalable, potentially revolutionizing medical education on a broader scale [18–20].

While our study provides compelling evidence for the benefits of 3D-printed models, it is not without limitations. The sample size was relatively small, and the study was conducted in a controlled environment with rigid polymer, which may not fully replicate the complexities of real-life clinical settings. Although Model 2 was derived from a different patient dataset, its normal geometry remained anatomically comparable to Model 1, and despite of that the assessment was performed on a separate day, some degree of procedural adaptation cannot be excluded. Future studies should employ anatomically distinct datasets to better evaluate pathological variations and to evaluate long-term skill retention. Future research should focus on larger, multicenter studies to validate these findings and explore the long-term impact of 3D-printed models on learning curve.

Additionally, further advancements in 3D printing technology could enable the creation of even more sophisticated models, incorporating elements such as tissue texture and dynamic blood flow. These enhancements would provide an even more realistic training experience, further bridging the gap between simulation and actual surgical procedures.

## Conclusion

Using 3D-engineering software and 3D-printing technology, anatomically accurate models of the aorta can be created. Combining with a structured training algorithm, it can enable inexperienced groups to become more familiar with basic navigation tasks. Adding to this the 3D-printed models cost-effective advantage over the other existing simulation options.

## Data Availability

The datasets used and analyzed during the current study are available from the corresponding author on reasonable request.

## Abbreviation

3D: Three-dimensional
EVAR: Endovascular aortic repair
